# Telerehabilitation Combined Speech-Language and Cognitive Training Effectively Promoted Recovery in Aphasia Patients

**DOI:** 10.3389/fpsyg.2018.02312

**Published:** 2018-11-22

**Authors:** Qiumin Zhou, Xiao Lu, Ying Zhang, Zhenghui Sun, Jianan Li, Zude Zhu

**Affiliations:** ^1^Department of Rehabilitation Medicine, The First Affiliated Hospital of Nanjing Medical University, Nanjing, China; ^2^Sucheng People’s Hospital, Suqian, China; ^3^School of Linguistics Sciences and Arts, and Collaborative Innovation Center for Language Competence, Jiangsu Normal University, Xuzhou, China

**Keywords:** speech-language training, cognitive training, aphasia, intervention, telerehabilitation

## Abstract

The present study investigated the efficacy of a computerized intervention for aphasia that combined speech-language and cognitive training delivered on an inpatient unit or via telerehabilitation to discharged patients. Forty inpatient and discharged aphasia patients were recruited and randomly assigned to the training group or control group. Computerized speech-language and cognitive training was provided for 14 days to the inpatients and 30 days to the discharged patients. Compared with the control group, training group had significantly more improved language function as assessed by the Western Aphasia Battery (WAB) and practical communication skills as assessed by the Communicative Abilities in Daily Living Test (CADL). It was also found that the positive effects of the computerized training when delivered via telerehabilitation to the discharged group were smaller than the effects when delivered on the inpatient unit. The results suggest that combining speech-language and cognitive training program is efficacious in promoting the recovery of patients with aphasia, both inpatients and discharged patients, and that the program works even when administered from a remote location.

## Introduction

Aphasia is an acquired language disorder due to brain damage, including damage due to stroke. The number of patients with aphasia caused by stroke has increased rapidly around the world (Engelter et al., 2006), with the prevalence estimated to be about 30–40% (Damico et al., 2010). Although some language functions are recovered a month after the stroke, nearly 60% of aphasia patients still do not fully recover after a year (Pedersen et al., 2004). Therefore, a large number of patients with aphasia need clinical speech-language rehabilitation training, which is a huge pressure on the current medical system, especially in China. One possible approach is to take advantage of telerehabilitation to facilitate speech-language rehabilitation. By delivering of rehabilitation services over telecommunication networks or the Internet, telerehabilitation could help patients far from the hospital or those at home in aphasia recovery.

Computerized training programs can largely benefit telerehabilitation in aphasia recovery (Hill and Breslin, 2016; Mallet et al., 2016). In fact, computerized speech-language training programs have been successfully applied in aphasia language rehabilitation (Palmer et al., 2012, 2013; Latimer et al., 2013). For example, by applying a computerized word-finding training program, persons with anomia significantly improved their naming performance (Fink et al., 2002; Doesborgh et al., 2004; Ramsberger and Marie, 2007) In addition to naming, recent studies have found that computerized speech-language therapy could improve speech-learning related performance in aphasia patients (Kurland et al., 2014). However, none of the above studies adopted a standardized scale to evaluate the training efficacy. One exception was the study by Des Roches et al. (2014). In this study, an iPad compatible speech-language training program was adopted in an aphasia intervention and the standardized Western Aphasia Battery-Revised (WAB-R) was used for efficacy evaluation. The researchers found that the computerized training program significantly promoted language rehabilitation in aphasia patients (Des Roches et al., 2014). However, the study recruited 42 subjects in the training group while recruited 9 subjects in the control group. It is unclear how the unbalanced subject number would affect the training effect comparison.

While the above cited aphasia therapies target speech-language pathology, recent theoretical developments and empirical evidence have suggested adding nonverbal cognitive training as part of speech-language recovery. In fact, in addition to speech-language impairment, aphasia is often accompanied by deficits in nonverbal cognitive abilities, including impairments in executive function, attention, and other skills (Seniow et al., 2009; Lee and Pyun, 2014; Marinelli et al., 2017). Some researchers have even suggested that improvement in nonverbal cognitive ability drives language recovery (Geranmayeh et al., 2014). Empirically, one of the most commonly used cognitive training methods is executive related training, including training in working memory and inhibitory control. Working memory training has significantly improved the performance of language comprehension in healthy elderly persons and patients with mild cognitive impairment (Mewborn et al., 2017). Yet only one study has used nonverbal cognitive training in speech-language rehabilitation of aphasia patients (Pei et al., 2015). The study found that attention training improved semantic processing but no phonological processing during word recognition in non-fluent aphasia. More importantly, they did not assess standard scales such as WAB. Thus it remains open for whether the training could improve general language ability.

This study investigated the efficacy of combined cognitive and speech-language training on aphasia recovery in both inpatient and discharged patients. Since the inpatient group was generally hospitalized for 2 weeks, a training time of 14 days was set. Considering that there was no on-site supervision of remote treatment (telerehabilitation) and there was no limit to the number of training days, we set a 30-day training time in the discharge group. If training was effective, then the computerized treatment in the inpatient training group (ITG) should be more effective than in the inpatient control group (ICG). Moreover, if the computerized training was effective, then the outcomes in the discharged patients’ training group (DTG) should be better than those in the discharged patients’ control group (DCG).

## Materials and Methods

### Participants

Forty patients with aphasia were recruited from Jiangsu Provincial People’s Hospital rehabilitation medicine center (22 males, mean age = 58.3 years, SD = 13.3) participated in the experiment, in which the duration of disease was 32.4 ± 18.6 days. The clinical diagnosis was cerebral infarction in 22 cases and cerebral hemorrhage in 18 cases. All participants were routinely treated with medications. Patients or their relatives signed the written informed consent form. No significant differences were found for age, duration of disease, gender ratio or etiology across groups (*ps* > 0.20).

Inclusion criteria were that there was no abnormality in language function before onset; patients were diagnosed with cerebral infarction or cerebral hemorrhage, and the lesions were stable; and the patients were able to perform training tasks, with no obvious memory impairment or intelligence impairment. Exclusion criteria were that patients had exacerbation, new infarctions or bleeding lesions, hearing impairments, visual disturbance, severe mental illness, or intellectual disturbance; could not tolerate the assessment tasks or treatment; or had epilepsy or disturbance of consciousness. For the characteristics and aphasia type category please see Tables 1, 2.

**Table 1:** Participants’ characteristics across groups.

Group	Age (years)	Duration (days)	Sex		Etiology	
			M	F	CI	CH
ICG	56.10 ± 17.29	29.90 ± 19.73	3	7	7	3
ITG	58.60 ± 11.44	34.80 ± 20.65	7	3	4	6
DCG	56.50 ± 14.34	32.80 ± 19.89	6	4	6	4
DTG	59.80 ± 11.26	31.00 ± 17.06	7	3	6	4

*ICG, inpatient control group; ITG, inpatient cognitive training group; DCG, discharge control group; DTG, for discharge cognitive training group, Duration, duration of disease; M, male; F, female; CI, cerebral infarction; and CH, cerebral hemorrhage.*

**Table 2:** Types of aphasia in the four study groups.

Group	BGA	TA	GA	BA	WA	AA	MTA	TCMA
ICG	1	0	4	2	0	3	0	0
ITG	2	0	4	2	1	0	1	0
DCG	0	1	5	1	0	0	2	1
DTG	1	0	5	3	0	1	0	0
Total	4	1	18	8	1	4	3	1

*BGA, basal ganglia aphasia; TA, Thalamic aphasia; GA, Global aphasia; BA, Broca aphasia; WA, Wernicke aphasia; AA, Anomic aphasia; MTA, Mixed transcortical aphasia; TCMA, Transcortical motor aphasia.*

### Training Protocol

Within the inpatient group and within the discharge group, participants were randomly assigned to the combined speech-language and cognitive training group or the control group, resulting in 10 participants in each subgroup. The ICG group was provided with routine treatment twice a day; the ITG group received the computerized speech-language and cognitive training. The DCG group engaged in family topics communication for 30 min a session, 2 times a day for 30 days, and the DTG group engaged in family topics communication for 30 min a day, with additional computerized speech-language and cognitive training, delivered via telerehabilitation, for 30 min a day for 30 consecutive days.

The telerehabilitation training program was adopted from the Wispirit Inc. (66nao.com). The training program included both a speech-language module and a cognitive training module. The training assignment was based on individual’s deficit profile. While language deficits can be standardized assessed by using WAB-R or other scales, it is clinically difficult to assess accompanied non-linguistic cognitive deficits in aphasia patients as they have difficulty in using language. To overcome such difficulty in a single study, the 66nao team has built up an association between cognitive and language deficits by using data driven approach by taking advantage of a large data bank. Therefore, in the present study, the language deficit profiles (see Table 3) assessed by WAB-R was used as the start point to assign language training tasks, and then the cognitive-language association was used to assign cognitive training tasks.

**Table 3:** Language deficits according to WAB-R for each aphasia type.

Aphasia type	Fluency	Repetition	Comprehension	reading	writing	naming
BA	×	×	Δ	×	×	×
WA	o	×	×	×	×	×
CA	o	×	Δ	×	×	×
GA	×	×	×	×	×	×
TCMA	×	o	o	Δ	×	Δ
TCSA	o	o	×	Δ	Δ	Δ
MTCA	×	Δ	×	×	×	×
AA	o	o	o	Δ	Δ	Δ

*o indicates normal performance; Δ indicates partial deficit; × indicates deficits.*

The speech-language module included training tasks on auditory comprehension, reading comprehension, repetition, naming, writing. Specific tasks mainly included words-picture association tasks, auditory digital span, sentence comprehension, working memory span based on Chinese words, sentence repetition, vocabulary repetition, picture naming for nouns and for verbs, picture-picture association naming, word-picture association naming, listening and writing for words and sentences, picture description based writing, synonym judgment, antonym judgment, word classification, consonant judgment, word meaning judgment, homophone error, tone judgment, non-word elimination, word construction.

The cognitive module included tasks about attention, memory, and executive function, which have been approved as effective approach for cognitive training in mild cognitive impairment population (Hill et al., 2017; Sherman et al., 2017) and healthy elderly (Lampit et al., 2014). Specific training paradigms included a paired-associate recall task, go-nogo task, Stroop task, Flanker task, switching task, attention span task and n-back working memory task.

To enable adaptive training, each task was designed with different levels of difficulty by adjusting the number of stimuli, the size of the stimulus, and the timing of the presentation. At the beginning, personalized assignment of speech-language tasks was mainly based on their WAB assessment and disease history, and assignment of cognitive tasks was similar across individuals. On each training day, the participant completed five cognitive rehabilitation games (2 min per day) and four speech rehabilitation games (5 min per day). Within each task, if there was high accuracy (> 80% of the norm in a typical aging population), the task was replaced by a harder task from the same domain. The training is thus adaptive at the participant level, with a similar setup but personalized progress across participants (Tang et al., 2016). The patients were required to completed the training tasks in hospital or at home by using personal computer or iPad kind of equipment.

### Outcome Assessment

Scores on the ten subscales of the Western Aphasia Battery (WAB; Shewan and Kertesz, 1980) are combined to form the aphasia quotient (AQ). In the current analyses, we used the AQ, as well as scores on fluency and content for spontaneous speech, auditory comprehension, repetition and naming, to assess speech-language ability.

The Communicative Abilities in Daily Living Test (CADL; Aten et al., 1982) requires the administrator to ask the patient 22 questions to assess a variety of communication skills. Across the 22 items, there are 34 sub-items, each scored from 0 to 4 points.

### Statistical Analyses

There were three parts of the statistical analyses performed with IBM SPSS 22.0. First, within the inpatient group and within the discharged group, *t-*tests were used to compare the training group and the control group on each dependent measure. Second, as the intervention of the control group was different for ICG and DCG, to overcome the unbalanced design, a 2 (Group: training group, control group) × 2 (Time: T1 for baseline, T2 for end of intervention) repeated measures analysis of variance (ANOVA) was conducted, with the AQ and CADL as the dependent variables to test the training efficacy. Then two 2 (Site: inpatient, discharged) × 2 (Group: training group, control group) × 2 (Time: T1 for baseline, T2 for end of intervention) repeated ANOVA were conducted, with the AQ and CADL as the dependent variables to test whether the training effect can be generalized from hospitalized aphasia to discharged aphasia. Third, as the AQ is a combination of the subscales, to avoid misconducting and in order to increase statistical power when testing a set of related dependent variables, we performed a 2 (Group: training group, control group) × 2 (Time: T1 for baseline, T2 for end of intervention) and a 2 (Site: inpatient, discharged) × 2 (Group: training group, control group) × 2 (Time: T1 for baseline, T2 for end of intervention) repeated measures multivariate analysis of variance (MANOVA), with the five WAB subscales of interest as the dependent variables.

## Results

To check on randomization, we used *t*-tests to compare the training group and control group on each of the dependent measures at baseline. The results showed that whether the participants were inpatient or had been discharged, there was no significant difference between the training group and control group on AQ (within inpatient group: *t*(18) = 0.77, *p* = 0.45; within discharged group: *t*(18) = 0.59, *p* = 0.56); five subscales of the WAB (within inpatient group: *ts* < 1.44, *p*s > 0.17; within discharged group: *ts* < 0.9, *p*s > 0.40); or CADL (within inpatient group: *t* (18) = 0.87, *p* = 0.40; within discharged group: *t* (18) = 0.43, *p* = 0.67).

Table 4 presents the evaluation scores before and after intervention. For the hospital group, the improvement in ICG and ITG was 14.1 and 26.5, for the discharge group, the improvement in the DCG and DTG was 7.1 and 19.8, respectively. Within each site, the two-ways ANOVA revealed significant main effect of Time (*ps* < 0.001) but not Group (*ps* > 0.75). A significant Group × Time interaction was significant was found (for inpatient group, *F*(1,18) = 12.22, *p* < 0.01; for discharge group, *F*(1,18) = 37.54, *p* < 0.001), showing larger improvement in the cognitive training group than the control group for both sites. Three-ways repeated measures ANOVA showed no significant three-way interaction (*F* < 1) or significant main effect of group or site (*Fs* < 1), but a significant main effect of Time (*F*(1,36) = 267.87, *p* < 0.001, T2 > T1) on AQ. Moreover, the Site × Time interaction was significant (*F*(1,36) = 11.20, *p* < 0.01), showing larger improvement in the inpatient group than the discharged group. There was also a significant Group × Time interaction (*F*(1,36) = 37.36, *p* < 0.001), showing that the training group had more improvement than the control group from T1 to T2.

**Table 4:** WAB and CADL performance in each subgroup (*M* ± *SD*).

DV	ICG		ITG		DCG		DTG	
	T1	T2	T1	T2	T1	T2	T1	T2
AQ	40.8 ± 27.3	54.9 ± 25.7	31.5 ± 26.5	58.1 ± 19.9	43.0 ± 30.7	50.1 ± 28.8	35.6 ± 25.5	55.3 ± 23.2
FL	3.6 ± 3.1	4.9 ± 2.7	2.6 ± 2.9	5.1 ± 2.4	3.8 ± 3.3	4.5 ± 3.1	2.9 ± 2.5	4.7 ± 2.5
CO	3.9 ± 2.8	5.7 ± 2.7	2.1 ± 2.7	4.8 ± 2.4	3.4 ± 3.3	4.0 ± 3.3	2.6 ± 2.8	4.7 ± 2.8
AC	5.4 ± 3.1	6.4 ± 2.8	3.8 ± 2.4	6.5 ± 1.6	5.0 ± 3.0	5.7 ± 2.7	4.6 ± 2.7	6.5 ± 2.4
RE	4.8 ± 3.2	6.2 ± 2.8	4.2 ± 3.2	7.0 ± 2.6	5.5 ± 3.0	6.3 ± 2.8	4.5 ± 2.4	6.7 ± 2.0
NA	3.5 ± 2.7	4.3 ± 2.5	3.2 ± 2.9	5.7 ± 2.3	3.7 ± 3.2	4.4 ± 3.0	3.2 ± 2.8	5.1 ± 2.4
CADL	24.2 ± 20.1	33.4 ± 21.7	16.6 ± 18.9	35.9 ± 21.8	25.3 ± 27.7	31.0 ± 28.8	20.4 ± 22.7	33.8 ± 23.4

*DV, dependent variable; AQ, aphasic quotient; CADL, communicative abilities in daily living test; FL, fluency; CO, content; AC, auditory comprehension; RE, repetition; NA, naming.*

The two-ways ANOVA results on CADL were similar to those on the AQ, within each site, there was significant main effect of Time (*ps* < 0.001) but not Group (*ps* > 0.75). A significant Group × Time interaction was significant was found (for inpatient group, *F*(1,18) = 6.36, *p* < 0.05; for discharge group, *F*(1,18) = 12.28, *p* < 0.01), showing larger improvement in the cognitive training group than the control group for both sites. Three-ways ANOVA across sites revealed that the scores increasing significantly after intervention, i.e., significant main effect of Time (*F*(1,36) = 108.57, *p* < 0.001). The main effect of Group (*F* < 1), the main effect of Site (*F* < 1), and the Group × Site interaction effect (*F* < 1) were not significant. However, the Site × Time interaction effect was significant (*F*(1,36) = 4.23, *p* < 0.05), with the inpatient group showing larger improvement than the discharged group. No significant three-way interaction was found among Site, Time and Group (*F* < 1).

Considering that the subscales from the WAB were related measures, repeated measures (MANOVA) was performed within each site (two-ways MANOVA) and across sites (three-ways MANOVA). Within each site, there was no significant Group effect (*Fs* < 1), but significant Time effect (*ps* < 0.001 for all five scores) and Group × Time interaction (*ps* < 0.001 for all five scores), while better performance at T2 than T1 in both the training group and control group was found, for each index the improvement in the training group was larger than the control group within each site (*ps* < 0.01). Then 2 (Site: inpatient, discharged) × 2 (Group: training group, control group) × 2 (Time: T1 for baseline, T2 for end of intervention) repeated measures (MANOVA) was performed, with the set of five subscale scores as the dependent measures. Consistent with the results for AQ, the results revealed no significant main effect or Group × Site × Time interaction (*p*s > 0.30), or Group × Site interaction (*F* < 1). There was significant Time effect (*ps* < 0.001 for all five scores), Group × Time interaction (*ps* < 0.001 for all five scores), while better performance at T2 than T1 in both the training group and control group was found, the improvement in the training group was larger than the control group. The Time × Site interaction was also significant for three of the five WAB subscales, namely fluency, content, auditory comprehension (*p*s < 0.05), but not repetition or naming, showing larger improvement in the training group than the inpatient group than the discharged group. The three-way interaction was not statistically significant (*F* < 1).

## Discussion

The purpose of this study was to test the efficacy of a computerized speech-language and cognitive training program for aphasia recovery. The program was delivered on an inpatient unit and via telerehabilitation to discharged patients. Our research had two main findings: first, combining speech-language and cognitive training improved the overall speech performance and daily communication skills in aphasia; second, the training program on the inpatient unit was also effective when delivered via telerehabilitation to the discharged group. Because of the randomized design and the use of multiple measures of improvement, these results provide strong evidence that computerized training in both speech-language and cognitive skills, whether implemented with inpatients or via telerehabilitation with discharged patients, can effectively improve aphasia recovery.

In line with previous results on inpatients (Fink et al., 2002; Doesborgh et al., 2004; Ramsberger and Marie, 2007; Des Roches et al., 2014; Kurland et al., 2014), this study demonstrated that computerized training could improve aphasia recovery while patients are in the hospital. It should be noted that both the control group and the training group showed at least some improvement from baseline to the end of the study, consistent with the view that aphasia patients have self-recovery, and traditional rehabilitation can help patients recover to some degree. Critically, inpatients’ improvement after computerized training was significantly higher in the training group than in the control group. The training effect was not only shown on the AQ, but also on several WAB subscales: spontaneous talking, auditory comprehension, repetition and naming. The results thus suggest that combined cognitive and speech-language training could improve general linguistic ability. Moreover, the training also significantly promoted performance on the CADL. The present study extended previous studies by assessing not just linguistic ability but also daily communication practice as indicators of aphasia recovery. The significant training effect, produced after 14 sessions, suggests that computerized training is an efficient training approach for patients and therapists during hospitalization.

More importantly, this study successfully delivered the computerized training program to discharged patients via telerehabilitation. Like the inpatient version of the program, the telerehabilitation program improved the recovery level of aphasia. The study found that the Site × Time interaction effect was significant, showing significant intervention improvement in inpatient group than discharged group on WAB and CADL. The key result was the significant Group × Time interaction, which did not significantly interact with Site. The results showed that, regardless of whether the participant was an inpatient or discharged patient, telerehabilitation training was more effective than routine intervention in promoting recovery. Interestingly, the inpatients group showed larger improvement than the discharged patients even though the training time was twice as long in the discharge group. This suggests that the inpatient intervention may have advantages over the discharged group. Given that age and the duration of disease were similar across groups, it is possible that the inpatient group was relatively more self-motivated for training than the discharged patients, which requires further clarification.

A significant characteristic of the training system used in this study is that it included not only speech-language training but also non-verbal cognitive training (e.g., working memory and attention training). Previous studies found that nonverbal cognitive training could improve the capacity to process Chinese characters (Pei et al., 2015). This study extended earlier results by showing that combined speech-language and cognitive training can promote the overall performance of language. The reason for how the combination could improve recovery may be that language and cognitive neural mechanisms are related. On the one hand, language processing itself needs cognitive participation (Zhu et al., 2011), and on the other hand, the improvement of cognitive ability itself is conducive to language use (Geranmayeh et al., 2014). Another benefit of multi-domain training is that it provides patients with a wealth of training programs to enhance enjoyment when engaged in repeated training.

This study had several shortcomings. First of all, the sample size was small; however, it should be noted that because of the no significant interaction among the three factors of group, site, and test time, there were actually 20 people per cell in the test of the group x time interaction (the core experimental test). Therefore, in general, this result was credible, and future research could recruit more participants to explore the effect of remote training. Secondly, an unbalanced design was used in the present study that the treatment was different in the ICG and the DCG. While the design may reduce the statistical power of the analysis, there was significant training effect showing larger improvement in the training group than the control group within each site. Future study may overcome the design and retest the training efficacy. Thirdly, there existed a certain degree of heterogeneity in the patients enrolled in this study, due to the small sample size and the large individual differences among patients with aphasia that can make rehabilitation training difficult. Generally speaking, large heterogeneity often leads to larger variance and smaller effect sizes. However, the inter-patient difference may be minimized by the adaptive training program. Specifically, the training difficulty was adaptively changed based on individual training curve, therefore the training program could be as best as possible to match each patient. Nonetheless, future research could make further efforts to provide training to different types of patients.

## Conclusion

The purpose of this study was to test the efficacy of a computerized training program for aphasia that included both speech-language and cognitive training, and to explore the impact of this type of training when administered to inpatients and to discharged patients in remote locations via telerehabilitation. It was found that, for both hospitalized patients and those who had been discharged, this combined form of computerized training promoted aphasia recovery more effectively than traditional training.

## Ethics Statement

This study was carried out in accordance with the recommendations of Ethic Committee of Nanjing Medical University with written informed consent from all subjects. All subjects gave written informed consent in accordance with the Declaration of Helsinki. The protocol was approved by the Ethic Committee of Nanjing Medical University.

## Author Contributions

QZ, XL, JL, and ZZ conceptualized and designed the study. QZ, XL, and YZ conducted the experiments. QZ, XL, YZ, ZS, and ZZ analyzed the data. QZ, JL, and ZZ drafted the manuscript. All authors were involved in the final manuscript revision.

## Conflict of Interest Statement

The authors declare that the research was conducted in the absence of any commercial or financial relationships that could be construed as a potential conflict of interest.
